# Simulated medication administration for vulnerable populations using scanning technology: a quasi-experimental pilot study

**DOI:** 10.1186/s12912-024-02248-6

**Published:** 2024-08-19

**Authors:** Anne Meginniss, Courtney Coffey, Kristen D. Clark

**Affiliations:** 1 Department of Nursing, University of New Hampshire, Durham, NH USA; 2 Department of Medical Sciences, Uppsala University, Akademiska sjukhuset, ingång 10, plan 3, Uppsala, 751 85 Sweden

**Keywords:** Simulation, Nursing education, Clinical education, Medication errors

## Abstract

**Background:**

Medication errors may occur due to shortcuts and pressures on time and resources on nurses. Nursing students are enculturated into these environments where their perceptions of norms around reporting and responding to medication errors are formative, yet simulated medication administration experiences are rarely reflective of the real-world environment. such as the standard use of medication scanning technology. The purpose of the present study is to test a pilot intervention, Medication Quick Response (QR) code scanning, and evaluate its effect on medication errors during simulation when compared to traditional simulation medication administration practices and to assess the students’ perceptions of the intervention.

**Methods:**

We conducted a quasi-experimental, observational study involving Junior and Senior (3rd and 4th year) undergraduate, pre-licensure nursing students from Spring 2022 until Fall 2023. Seven simulations were conducted in pediatric and obstetric courses. The intervention group used non-patented, low cost QR scanning during medication administration. The control group used standard manual administration. Medication errors were measured based on the quantity, type of error, and degree of patient risk. A Qualtrics survey was used to assess the students’ perceptions of the intervention following simulation participation.

**Results:**

A total of 166 students participated in the study. In each course, 7 groups were assigned to the intervention and 8 were assigned to the control. More than half of the groups made at least one medication error (*n* = 17), one-third of groups (*n* = 10) made a high-risk medication error. There was no statistically meaningful difference in the rate, type, or potential patient risk of medication errors between the intervention and control groups. The majority of participants (*n* = 53) felt that QR scanning more closely mimicked medication administration in clinical settings. Half of the participants responded that it improved their safety practices (*n* = 37).

**Conclusions:**

The results of this pilot study indicate that while there is a high risk for error among pre-licensure nursing students, the use of QR scanning did not increase the risk of medication errors. The next study iteration will build upon these pilot findings to integrate the use of embedded medication errors, time management tasks, and a multi-site implementation.

**Supplementary Information:**

The online version contains supplementary material available at 10.1186/s12912-024-02248-6.

## Introduction

The Academy of Managed Care Pharmacy (AMCP) estimates that medication errors harm 1.5 million patients per year in the United States (US) and are responsible for up to 98,000 patient deaths annually [1]. Medication errors pose significant risks to patient safety and are a prevalent concern in healthcare settings worldwide. Nurses play a pivotal role in medication safety as they are the primary healthcare professional who administers medication, making them the final line of protection between patients and medication errors. Before the COVID19 pandemic, the global nursing workforce was already considered well below demand, with projected growth insufficient to meet healthcare system needs due to the retirement of existing nurses and an aging population [2]. During the COVID19 pandemic, an unprecedented workforce strain occurred, with health systems becoming overwhelmed and healthcare staff contracting COVID19 at high rates, some of whom experienced post-COVID syndrome which further impeded their ability to return to work at full capacity [3, 4] The increasing healthcare system strain, chronic understaffing, pressures to meet increasingly acute patient needs, and throughput metrics have resulted in challenges to nurses’ ability to safely administer medications.

Medication safety practices are a cornerstone of nursing education, and students are ingrained with the 5 rights of medication administration to reduce the risk of such errors. These rights include: right patient, right drug, the right dose, the right route, and the right time. However, this approach has long been critiqued for lacking the specificity and depth to accurately depict the complexity of medication errors [5, 6]. An error can occur at many different points in the administration continuum. Specifically, they can occur at the time a medication order is placed, during its preparation with the pharmacy, when the nurse retrieves the medication from the medication dispensing system, when the nurse is preparing the medication to be delivered, or during its administration to the patient. In addition to the error’s place in the medication administration continuum, there is variation in the types of medication errors that can occur. The most common types of medication errors observed in hospital settings are timing errors (early or late doses), omission errors (doses missed), and dosage errors (too small or too large; [7, 8]). High-profile cases of medication errors resulting in significant patient harm have entered the public consciousness and, in one instance, resulted in the rare occurrence of a nurse receiving criminal charges in the US [9, 10]. While conversations across healthcare have taken place around the relationship between patient safety and strained staffing environments, there has been less discussion surrounding the enculturation of prelicensure nursing students to normalized workarounds and shortcuts in patient care delivery.

Medication safety is a primary component of nursing education. However, witnessing senior nurses or preceptors engaging in workarounds or substandard medication safety practices normalizes these practices for pre-licensure and recently graduated nurses [11]. Novice nurses may not have the fortitude to question a senior coworker’s practice given the power gradient that exists between them [12]. It is difficult to know the exact rate of medication errors made by recently graduated nurses, as this number is likely underreported, however, a survey found that 55% of recent graduate nurses reported having made a medication error [22]. Additionally, a study of pre-licensure nursing students using simulation found that by the end of four semesters, 80% of participants were not engaging in safe medication administration practices [13]. It is possible that this high rate of unsafe medication administration practices observed in the study could be due to a lack of realism in simulated medication administration [14], leading students to perceive the activity as having little impact on real-world patient care. This suggests a need for recurrent education and evaluation of safe medication practices throughout the curriculum with an emphasis on the replication of real-life practices.

Studies have demonstrated that nurses spend approximately 40% of their time at work focused on medication management [15]. It is critical to examine whether adequate time is spent on education surrounding medication safety and, more importantly, to ask if the education provided is effective. Simulation has been used to create learning opportunities for nursing students to practice medication administration. Engaging in medication administration during high-fidelity simulations has been shown to increase nursing student’s knowledge related to medication safety [16]. However, simulation has been described as limited in its capacity to incorporate all the features of real clinical settings. The aspect of realism is a key component of fidelity and one of the cornerstones of evidence-based simulation practice [14, 17]. Realism in simulation, through physical, emotional, and psychological approaches, is associated with higher competency evaluations and engagement among nursing students. Yet realism in application to medication administration has lagged behind clinical practices in favor of more manual approaches. Current practices rely heavily on memorization and do not use resources commonly available in clinical settings. For example, students are often tasked with preparing large numbers of notecards with extensive details on each medication that is assigned to each patient [18], yet integration of currently available technology, such as smartphones or medication barcode scanning, is underutilized. Such technologies have been shown to have educational benefits, such as improved collaboration with peers and mentorship in clinical settings [19], as a tool to deliver medication administration information [20, 21], and an increase in perceived clinical preparedness [22, 23]. Incorporation of technology that is readily available in clinical settings into simulation provides a pathway for students to investigate the realities of practice and explore technology’s potential limitations in a controlled setting.

Incorporating real-life tools into the medication administration process offers the opportunity for students to identify the limitations of that technology and to encounter circumstances where they must question the accuracy, or respond to irregularities as they arise, thereby increasing their capacity for critical thinking and adaptation in real-world environments. However, the software and tools required to integrate this technology into educational simulation settings are costly, inefficient, partially functional, or incompatible with existing technologies. Therefore, the purpose of the present study is to test a pilot intervention, Medication Quick Response (QR) code scanning, and evaluate its effect on medication errors during simulation when compared to traditional simulation medication administration practices with pre-licensure, undergraduate nursing students. We will also evaluate the students’ perception of the intervention for future implementation in a larger-scale study.

## Materials and methods

### Recruitment and procedures

A quasi-experimental, observational study was used to address the present aims. Junior and Senior prelicensure nursing students enrolled in pediatrics and obstetrics courses from Spring 2022 until Fall 2023 were recruited to participate. Pediatrics and obstetrics courses were chosen for this intervention’s pilot because medication errors are particularly concerning in the context of these vulnerable populations [8, 24]. Medication administration for these populations can be particularly complex and an error can have more severe effects on children, pregnant persons, and fetuses.

The course simulation sections for pediatrics and obstetrics courses were assigned as either control or interventions based on alternating weeks and where they were placed during course registration. Groups registered to week A were placed in the control, and groups registered to week B were placed in the intervention. Simulation groups were comprised of four to six students who worked through the simulation scenario collaboratively. Medication errors were subsequently analyzed based on group performance as opposed to individual participants.

### Before the simulation

Before the simulation, students were provided with preparation materials, which included access to the patient chart and protocols that were utilized during the scenario. Medications that were available during the simulation were listed and students had the opportunity to look up medications before and during the scenario. A guided preparation homework assignment was completed by all students before the simulation. The purpose of this assignment was to review content from the course and to highlight important information that would be addressed within the simulation. Once the students arrived at the lab on the day of the simulation, a pre-brief was completed. During the pre-brief, the scenario was explained in further detail and roles were assigned. Students had the opportunity to ask questions before the initiation of the simulation.

### Simulation scenarios

The simulation scenarios used in this study were developed per the INACSL Standards of Best Practice [17, 25]. In the obstetrical health course, two high-fidelity simulation scenarios were used involving medication administration to mother and baby dyads (i.e., Brenda and Renee). In the pediatrics course, five high-fidelity simulation scenarios were used involving medication administration (i.e., Sam, Sabina, Jack, Charlie, and Abigail). All simulation scenarios took place in a simulated inpatient hospital setting with a laptop that provided access to the patient’s chart and medication administration record through DocuCare [26]. Participants engaged with one scenario in each session. In the intervention groups, the patient wristband and medications for the simulation were labeled with barcodes. In the control groups, standard manual administration processes were followed (i.e., no barcodes were provided on medications or patient wristbands). All groups had access to the simulation case, including medications, before participating in the scenario. The simulation scenarios contained between three and six medications to be administered. All scenarios except one (Charlie) contained at least one high-complexity medication to be administered (e.g., ceftriaxone to be administered piggybacked to intravenous fluids which requires drug compatibility verification, dosage calculation, and to program the pump). A full description of the medications by scenario and complexity can be found in Supplemental Table 2. No medication errors were embedded into the scenarios.

### Measurement

#### Demographics

Participants were asked to indicate which academic year they were currently enrolled in (i.e., Junior or Senior). Participants were also asked whether they had previous experience with medication administration using scanning technology, indicating yes or no.

#### Intervention

Current tools available for nursing education pose barriers to the broad implementation of medication administrative technologies representative of real-world clinical practice. Electronic health record systems developed for nursing education are cumbersome and labor-intensive to integrate medication and wristband scanning capabilities. Alerts for mis-scanning medications are unreliable, providing a false sense of safety and difficult for instructors to observe the information the students are receiving. The programs are also costly, a challenge for many nursing programs to obtain. For the present study, Medication Quick Response (QR) code scanning was tested as a non-patented, low-cost method to replicate traditional medication barcode scanning in the simulation setting. QR codes were created using a free QR-generating website [27] and placed on the patient’s wristband and medications used within the simulation scenarios YouTube shorts videos were created by study personnel for use during the simulation (Supplemental Table 1). Upon scanning the barcode on the patient identification band with their mobile phone, a YouTube short video displaying the patient chart played. When the participants scanned the medication barcodes, another YouTube shorts video was played with medication information. Students also could view the medication order and chart using the provided laptop in the patient’s room. The control groups did not receive barcode scanning and administered medication per standard processes.

#### Medication errors

Faculty members oversaw each simulation group to assess for medication errors. Medication errors were measured by quantity, type of error, and category of potential patient risk. The types of medication errors were defined based on the U.S. Food and Drug Administration’s healthcare professional reporting form [28] and included: compatibility error, incorrect administration technique, incorrect administration time, known allergy, reconstitution error, wrong dose, wrong medication, wrong patient, wrong route, and unsafe to administer. Please see Table 1 for the full list of error types and their operational definitions. After making a medication error, faculty used the National Coordinating Council Medication Error Reporting and Prevention (NCC MERP) Index to determine the potential patient risk imposed by the error. The NCC MERP assigns an ordinal range of categories (A = near miss event, to I = error causing/contributing to patient death) to medication errors. This index is intended for use in clinical settings with real patients, therefore we used the following categories and numbered them from 1 to 4: A- near miss, B- error did not reach patient, C- reached patient, but no harm, D- patient required monitoring or intervention, but no significant harm). This variable was dichotomized as low potential patient risk (less than 3) and high potential patient risk (3 or more).

**Table 1:** Medication error types and their definitions as applied during evaluation of medication administration during pediatric and obstetric simulation scenarios

Error Type	Definition
Compatibility Error	This occurs when two or more medications are administered together and are designated as incompatible due to the risk of adverse reactions to the medication or patient. For example, ceftriaxone and lactated ringers are not compatible.
Incorrect Administration Technique	This occurs when a medication is intended to be delivered at a certain rate but is administered differently. For example, medication should be infused at the Y site on the intravenous line but is piggybacked on the primary infusion instead.
Incorrect Administration Time	This occurs when the medication is administered incorrectly based on the dose frequency of the prescribed medication. For example, acetaminophen was administered 4 h after the last dose, but it was ordered every 6 h.
Known Allergy	This occurs when a medication is administered to a patient who has a known allergy to the medication.
Reconstitution Error	This occurs when the nurse mixing the medication, performs a mixing error affecting the concentration that alters the intended dose. For example, mixing ceftriaxone for intramuscular administration based on the requirements for intravascular administration.
Wrong dose	This occurs when a medication is given that is above or below the correct dose for the patient based on the dose range. For example, 2 mg of morphine was administered, but the ordered dose was 0.5–1.5 mg.
Wrong Medication	This occurs when an inappropriate or incorrect medication is administered that was not prescribed. For example, the patient was prescribed hydroxyzine but was administered hydralazine.
Wrong Patient	This occurs when a medication is administered to a patient for whom it was not prescribed.
Wrong route of Administration	This occurs when the medication is administered a different route than intended based on the order or by the manufacturer. For example, a flu vaccine was given intravascularly instead of intramuscularly as intended by the manufacturer.
Unsafe to Administer	This occurs when a medication is given as prescribed but under symptoms or conditions that can cause harm to a patient. For example, administering a beta-blocker to a patient whose heart rate is 30.

##### Students’ perceptions

Participants were provided a brief Qualtrics survey that asked three questions about their perceptions of the intervention. Students were asked, “If you used the QR code scanning, did you feel it improved your medication administration during simulation?” with a 3-point Likert-type scale for responses (i.e., yes, maybe, and no). Participants were also asked, “If you used the QR code scanning in simulation, do you feel it simulated medication scanning in the clinical setting?” with a 3-point Likert-type response. Students were also asked “Do you think the QR code scanning prevented (or could have prevented) a medication error during the simulations?“ with the same response options. Lastly, an open-text response question was provided, asking students to provide further feedback on their experiences in the simulations.

### Analysis

Descriptive statistics were used to describe the characteristics of the simulation groups and observed medication errors. Mann-Whitney U-tests were used to analyze data for differences between intervention and control groups with a significance threshold of p <. 05. Open-text responses related to students’ perceptions of the simulations were analyzed using thematic analysis [29]. Two research personnel evaluated responses and coded inductively. Initial codes were then evaluated to identify final themes.

## Results

A total 178 (32 groups) students completed simulation scenarios in obstetrics and pediatrics courses across two semesters. Initially, there were eight intervention and eight control groups per course. Two intervention groups were dropped from the final analysis, one from each course, because of declined informed consent. A total of 166 students, divided into 30 groups, were included in the final sample. Seven groups in each course received the intervention, with the remaining eight acting as controls for a total of 14 intervention groups and 16 controls.

### Medication errors

Approximately 56% (*n* = 17) of groups made at least one error. The operational definitions can be found in Table 1. The most common error observed was incorrect medication administration technique (53.3%, *n* = 22). One-third of participants (*n* = 10) made an error that posed a high potential patient risk. Differences between control and intervention groups regarding the rate of errors, type of errors, and risk potential were examined (Table 2).

**Table 2:** A description of the number of errors and the results of Mann-Whitney U-tests for differences in the number of medication errors (intervention = I, control = C), type, and error risk between intervention and control groups

Medication Errors		Number of Errors		z	*p*
		I	C		
*Among All Simulations*		14	16		
Difference in the number of medication errors				0.197	0.844
Difference in error type^a^					
	Incorrect administration technique	10	12	0.677	0.500
	Reconstitution error	1	1	-0.141	0.888
	Wrong dose	1	2	-0.211	0.833
	Wrong medication	1	1	-0.480	0.631
	Wrong route	1	0	-1.069	0.285
Difference in error category				0.649	0.517
*Among Pediatrics Simulations*		7	8		
Difference in the number of medication errors				-0.151	0.880
Difference in error type					
	Incorrect administration	6	8	0.500	0.617
	Reconstitution error	0	0	No observations	-
	Wrong dose	0	0	No observations	-
	Wrong Medication	0	0	No observations	-
	Wrong Route	1	0	-1.069	0.285
Difference in error risk				-1.069	0.285
*Obstetrics Simulations*		7	8		
Difference in the number of medication errors				0.605	0.546
Difference in error type					
	Incorrect administration	4	4	0.513	0.690
	Reconstitution error	1	1	-0.198	0.857
	Wrong dose	2	2	-0.299	0.765
	Wrong medication	0	1	0.500	0.617
	Wrong route	0	0	No observations	-
Difference in error risk				1.292	0.196

^a^Only the error types observed at least once are reported

No statistically meaningful difference in the number, type, or potential patient risk of medication errors between intervention and control groups was observed (Table 2). Further analysis was performed to examine group differences within each course (i.e., obstetrics and pediatrics), and found no statistically meaningful differences in the number, type, or potential patient risk of medication errors between intervention and control groups (Table 2).

#### Students’ perceptions

Sixty-six students (40.9% response rate) completed the survey assessing their perceptions of the intervention at the end of the semester. Fifty-nine (86.7%) students had previous experience using QR code scanning technology. When asked if they felt QR code medication scanning improved their medication safety practices, 54.4% responded “yes” (*n* = 37). Approximately three-quarters (*n* = 53) felt, “yes, it simulated” or “somewhat simulated” medication scanning in the clinical setting. When asked “do you think the QR code scanning prevented (or could have prevented) a medication error during the simulations?”, 85.9% (*n* = 49) responded “yes” or “somewhat.”

### Students’ perceptions: qualitative

Students were provided an open-text box to provide further feedback on their experiences in the simulations. Responses were grouped into themes: *replicated realistic clinical medication scanning* and *supported critical thinking*.

#### Replicated realistic clinical medication scanning

This theme was characterized by students describing similarities between the intervention and clinical medication scanning. One student stated, “this was a great simulation experience! QR codes felt more like real life and have double checks.”

#### Supported critical thinking

This theme was characterized by students describing how the simulation with medication administration improved their critical thinking related to medication administration due to technology. One student stated, “I think scanning helped us think through the medication administration before we did it.”

## Discussion

The purpose of the present study was to test a pilot intervention, the implementation of medication administration technology (e.g., medication barcode scanning), and evaluate its effect on medication errors during simulation when compared to traditional simulation medication administration with pre-licensure, undergraduate nursing students.

It was found that the implementation of QR code medication scanning during simulations was not statistically different in terms of error rates, types, or potential risk to patient safety when compared to traditional simulation of medication administration. While improved medication safety was not observed, there was no increased risk of error, suggesting that the incorporation of QR scanning should be explored for inclusion as standard practice in simulation settings.

The study also aimed to evaluate students’ perceptions of this intervention for future implementation in a larger-scale study. Most students had experience with medication scanning before the simulation, indicating that the use of medication scanning in the simulation would align with real-world clinical environments. Yet, the lack of integration is evidence of a considerable gap between simulation experiences and clinical settings. These participant perspectives further support the urgency to bridge differences between the two environments. The results of this study provide support for the use of Medication Quick Response (QR) codes as a low-cost, efficient, and accessible tool to accomplish this goal. Additionally, this study exhibits how realism can be improved through the use of medication scanning technologies [21, 23], even with limited resources.

Within the open-text responses obtained from participants at the end of their participation in the study, the use of QR code scanning to simulate medication administration barcode scanning was perceived to encourage critical thinking. However, participants did not specify *how*. It is possible that students perceived this use of critical thinking in relation to the medication complexity and not the scanning itself. Alternatively, this could be related to the standardization of medication administration safety practices and the simulation of students’ real-world experiences where they are required to utilize critical thinking in medication administration broadly.

### Limitations

While the present study was a pilot, there are limitations to note. The sample size was relatively small and from a single study site, and therefore, is not representative of the pre-licensure nursing student population. Additionally, there was a lack of randomization and no allocation concealment within the study. The simulation scenarios also did not include embedded errors, which would mimic real-world applications. An example of this would be purposefully having a barcode that did not scan. The utilization of embedded errors would further the utilization of critical thinking skills to determine appropriate next steps.

### Next steps for implementation

The current pilot study indicates that the inclusion of QR scanning during simulation medication administration did not increase the risk of medication error, providing foundational information which the next study iteration can build from. A feasibility study is planned to advance the findings of the present study through the addition of embedded medication errors (e.g., barcodes that do not scan, barcodes that scan as the wrong medication), time management evaluations for polypharmacy (e.g., on-time parameters, medication prioritization), and questioning individuals of actual or perceived authority (e.g., provider, pharmacist). Embedded distractions, such as a family member in the room asking questions during medication administration, would also increase complexity in a manner that mimics real-world care environments and factors that increase the risk of medication errors [23] and are also a feature that is planned for feasibility testing.

With these added layers of complexity, an assessment of feasibility and the appropriateness of scaling these features to students’ level of learning and skills is needed. Through feasibility testing of embedded features within simulated medication administration, a recommending scaling approach can be developed based on students’ level of education and experience, but also integrated across the nursing curriculum.

### Future directions

As nursing education advances, simulation offers a unique opportunity for the development of skills but also for the mindset of future practicing nurses. Paulo Freire described the role of oppression and learning, where students are in positions of little power in their schools and universities, an experience that carries through to the clinical settings for nursing students [30, 31]. Shifting the view of nursing students and novice nurses as inferior, passive recipients of knowledge to active participants who are capable of creating a culture of change allows for enriched, meaningful learning experiences built on the foundation of critical thinking skills that can carry into complex clinical experiences, such as medication administration. This can be accomplished through future research investigating increased complexity in medication administration and by reimagining how outcomes for the simulations are defined.

In addition to the steps outlined as planned for implementation following this present study, additional approaches to integrating medication administration complexity include the use of multiple patient simulations occurring at the same time to expose students to the administration and prioritization of multiple medications [22]. Research focused on understanding students’ reliance on scanning technology when administering medications would inform areas where complexity can be enhanced and reliance on technology can be challenged [32, 33]. Integration of interpersonal professional scenarios forms another opportunity for medication administration complexity development. Examples of interpersonal professional scenarios could include circumstances requiring students to question the safety or appropriateness of medication orders or a situation where students must respond to an observed medication error or safety concern made by a colleague.

The use of simulation as a tool to empower and advance critical thinking calls for a reimagining of successful simulation outcomes. Evaluation of simulation performance often uses a pass/fail approach, where students fail if they make a medication error. However, medication near misses and errors will occur in the clinical setting. Expanding the definition of success to include an appropriate response to a medication error, such as reporting and documentation, would aid students in learning how to respond to such events and enculturate them to a process improvement practice philosophy. These varying approaches, from complexity to expanding the definitions of success, could be studied toward the development of an operationalized approach to simulation medication administration across the spectrum of nursing education so that medication admiration experiences can be scaled by difficulty and level of learning, integrated across cohorts and programs.

## Conclusion

The present findings reveal a concerning prevalence of errors, even amongst the groups that utilized the QR code medication scanning method. The use of QR scanning during simulated medication administration was, however, found to have no difference in medication errors when compared to standard practices. Participants reported that the use of QR scanning more closely resembled their clinical settings and that they perceived a greater use of critical thinking skills. This pilot provides initial evidence that medication barcode scanning can be implemented with low resource tools and should continue to be explored for implementation across nursing curricula in a movement toward greater realism in nursing simulation related to medication administration practices. These findings underscore the importance of advancing simulation practices to address medication errors within nursing education, particularly in highly complex patient populations such as pediatrics and obstetrics.

### Electronic supplementary material

Below is the link to the electronic supplementary material.

Supplementary Material 1

## Data Availability

The datasets generated and/or analysed during the current study are not publicly available due conditions of the data management agreement to protect participant privacy. The data that support the findings of this study are available upon reasonable request from the authors.
