# Supplementary material for: Simulated medication administration for vulnerable populations using scanning technology: a quasi-experimental pilot study
Source: BMC Nurs. 2024 Aug 19;23:576. doi: 10.1186/s12912-024-02248-6 (PMC11334313; doi:10.1186/s12912-024-02248-6)
Supplement: Supplementary file 1 — Supplementary Material 1 [file 12912_2024_2248_MOESM1_ESM.docx]

Supplemental Table 1. QR Code Medication Scanning High-Fidelity Simulation Road Map, Example Case.

| Patient History | Patient Name: Samuel Jones  Age: 7 week-old (49 days)  Gender: Male  Weight: 3.9 kg  Allergies: NKDA  Dx: Fever unknown origin  Sx: 7-week-old infant who presents to the emergency department with cc: fever. Father (caregiver) reports 1 day history of fussiness, decreased oral intake (similac) and decreased number of wet diapers. Father(caregiver) reports difficult to tell if infant has urinated because the diapers are mixed with stool, but the quantity is less than 2-3 days ago. His Tmax was 100.8 at home rectally 1 hour prior to arrival.  He has no known medical problems. Vaccinations are up to date and NKDA.  No sick contacts at home. | | |
| --- | --- | --- | --- |
| Medications Ordered | Medications: (route; dose/rate)   1. Normal Saline IV (stat) 2. Acetaminophen oral (PRN) 3. Ceftriaxone IV (stat)   Barcodes for intervention group directed to the following links:  Wristband: https://youtu.be/MfitxHP-OWA  Medications: https://youtu.be/R58jymM4pQ8 | | |
| Initial Assessment | *Initial Vital Signs*:  HR**:** 144 ,SpO2:97% RA,  BP: 88/45,  RR: 40  Temp: **101.5 R (38.6 C)**  *Assessment Findings:*  Airway: patent  Lung Sounds: clear, no distress  HEENT: Anterior fontanel bulging  Heart Sounds: normal  Breathing: normal  Patient Personality/Mood: normal  Mental Status: at baseline  Skin: pink, warm, dry  Other: Parent at bedside | *Ensure patient safety* 🡪  Place on monitor  *Assessment* 🡪perform assessment on infant. 🡪  Student should use Children Hospital of Philadelphia (CHOP) algorithm to support assessment.  *Check a blood glucose*. Based on presentation. 🡪  Perform family center care. 🡪 | *Patient Safety*- Proper patient ID, hand washing, precautions.  *Assessment*- Should verbalize assessment findings to support assessment.  *Recognition*- Recognize of significant risk of fever in this population and possible hypoglycemia.  *Family Centered Care*- discuss findings with mother, explain procedures and ensure she understands.  *Critical thinking*- Pt is 49 days old and should choose the appropriate algorithm based on age. |

Supplemental Table 2. A description of the medication orders per scenario, their characteristics, and their degree of complexity/difficulty based on expected skill level.

| **Scenario** | **Discipline and Topic** | **Ordered Medications**^a^ | **Route** | **Reconstitute** | **IV Compatibility** | **Calculate Dosing** | **Program Pump** |
| --- | --- | --- | --- | --- | --- | --- | --- |
| Samuel | Pediatrics: Febrile Infant | Acetaminophen | PO | No | N/A | Yes | N/A |
|  |  | Ceftriaxone | IV | Yes | Yes | Yes | Yes |
|  |  | 0.9% Normal Saline bolus | IV | No | Yes | Yes | Yes |
| Sabrina | Pediatrics: Asthma | Albuterol | Nebulizer | No | No | Yes | No |
|  |  | Ipratropium | Nebulizer | No | No | Yes | No |
|  |  | Prednisone | PO/IV | No | Yes | Yes | Yes |
|  |  | Dexamethasone | PO | No | No | Yes | No |
|  |  | Magnesium Sulfate | IV | No | Yes | Yes | Yes |
|  |  | 0.9% Normal Saline Bolus | IV | No | Yes | Yes | Yes |
| Jack | Pediatrics: Seizure | Ativan | IV | No | Yes | Yes | No |
|  |  | Levetiracetam | IV | No | Yes | Yes | Yes |
|  |  | Fosphenytoin | IV | Yes | Yes | Yes | Yes |
|  |  | Midazolam | IV/IN | No | Yes | Yes | No |
| Abigail | Pediatrics: ALL fever | Acetaminophen | NG | No | No | Yes | No |
|  |  | Ceftriaxone | IV | Yes | Yes | Yes | Yes |
|  |  | 0.9% Normal Saline bolus | IV | No | Yes | Yes | Yes |
| Charlie | Pediatrics: Anaphylaxis | Epi Pen Jr. | IM | No | No | Yes | No |
|  |  | Diphenhydramine | IV | No | Yes | Yes | Yes |
|  |  | 0.9% Normal Saline | IV | No | Yes | Yes | Yes |
|  |  | Methylprednisolone | IV | No | Yes | Yes | Yes |
| Brenda | Obstetrics: Labor and Delivery | Lactated ringers | IV | No | No | No | No |
|  |  | Penicillin | IV | No | Yes | No | Yes |
|  |  | Pitocin | IV | No | Yes | No | Yes |
| Renee | Obstetrics: Postpartum Hemorrhage | Methergine | IM | No | No | No | No |
|  |  | Hemabate | IM | No | No | No | No |
|  |  | Misoprostol | PO or rectal | No | No | No | No |
|  |  | Tranexamic Acid | IV | No | Yes | No | Yes |
|  |  | Pitocin |  |  |  |  |  |
| ^a^Medications are color coded by complexity/difficulty: green = low, yellow = moderate, red = high | | | | | | | |
